# Corporate social responsibility: A Driver for green organizational climate and workplace pro-environmental behavior

**DOI:** 10.1016/j.heliyon.2024.e38987

**Published:** 2024-10-05

**Authors:** Sara Kanwal, Abdullah Al Mamun, Mengling Wu, Saad Mahmood Bhatti, Mohd Helmi Ali

**Affiliations:** a UKM - Graduate School of Business, Universiti Kebangsaan Malaysia, 43600, Selangor Darul Ehsan, Malaysia; b University of Engineering and Technology, Lahore, Punjab, 39161, Pakistan

**Keywords:** Corporate social responsibility, Pro-environmental behavior, Green organizational climate, Green shared vision, Social exchange theory, Social identity theory

## Abstract

In line with the United Nation's sustainable development goals (SDGs), the unsustainable use of scarce natural resources and worldwide environmental degradation call for the immediate implementation of green behaviors across all organizations. As environmental issues originate from human activities, they necessitate the realization and execution of employees' workplace pro-environmental behaviors (WPEB) and their stimulators. Drawing on social exchange and social identity theories, this study first investigates the mediating role of green organizational climate in the relationship between various forms of perceived corporate social responsibility (CSR) and employees' WPEB. It further examines the mediated moderation via green shared vision. An adapted survey questionnaire was distributed to senior-level and middle-level managers of large and medium-sized manufacturing firms in Pakistan, and 349 responses were gathered. The partial least-squares technique was used for data analysis. The findings indicate that all forms of perceived CSR positively correlate with a green organizational climate, which further significantly leads to employees' WPEB. The results also show that a strong shared green vision among employees can improve the relationship between the green organizational climate and employees' WPEB and vice versa. Moreover, multi-group analysis shows that the relationship between customer-centric perceived CSR and green organizational climate was stronger for medium-sized firms than large ones. Thus, to ensure the implementation of SDGs such as decent work and climate action, firms' top management must recognize the importance of investing in both internal and external CSR initiatives to foster a green organizational climate leading to employees' WPEB. Companies should communicate their participation in CSR activities to all stakeholders through public platforms to increase inspiration. Policymakers should further introduce CSR excellence awards and non-compliance penalties to encourage firms' extensive participation.

## 1 Introduction

The rapid pace of economic development is accompanied by detrimental practices, such as the overexploitation of natural resources and the generation of substantial waste. These activities have given rise to a plethora of environmental issues [1] that not only impede national progress and economic growth but also pose significant risks to human health and safety [2]. Since climate change has become an urgent global issue, climate action failure and resource scarcity also rank among the top global risks [3]. In support, the United Nations (UN) proposed sustainable development goals (SDGs) such as (Goal 8) decent work, economic growth, and (Goal 13) climate action [4]. COP26 in 2021 further highlighted widespread global attention towards increasing environmental issues and the urgency to address and resolve them [5]. Consequently, governments have pledged to inculcate and adopt greener business strategies to promote sustainable behaviors. Specifically, the manufacturing sector is recognized as a primary contributor to pollution in both developed and developing countries, necessitating a thorough evaluation, monitoring, and appropriate modification of its managerial responsibilities [6,7]. Pakistan's manufacturing industry is a major user of natural resources and a key contributor to environmental degradation [8]. According to the Pakistan Bureau of Statistics, the manufacturing sector's production was estimated to tremendously increase by 8.7 % in 2021, making it the largest gain over the previous five years [9]. Conversely, Pakistan's Global Environmental Performance Index score was only 24.6, ranking 176th among 180 countries [10].

Therefore, manufacturing organizations, specifically in Pakistan, require the establishment of sustainable pro-environmental initiatives [11]. Environmental issues stem from human economic activities [12], making it imperative to change human behavior and practices to lessen their detrimental impact on the environment [7]. The environmental advantages resulting from contemporary scientific and technological advancements fall short of compensating for the inflicted environmental harm. Consequently, to effectively address environmental degradation and achieve sustainable development, organizations must prioritize strategies related to their internal operations and practices [13]. This necessitates an examination of managerial practices that lead to the adoption of environmentally friendly attitudes and behaviors among employees. Organizations may enforce environmentally sustainable standards through stringent regulations [14]; however, the success of most green initiatives ultimately relies on employees’ involvement in green behaviors, known as workplace pro-environmental behaviors (WPEB) [11,15]. These behaviors encompass activities such as recycling, conserving energy when not in use, and actively supporting environmentally responsible practices promoted by the organization [16].

Myriad studies highlight that to promote employees' WPEB, organizations should embrace the micro-level corporate social responsibility (CSR) phenomenon [17,18]. One pivotal approach involves understanding employees' perceptions of these initiatives [19]. Employee-perceived CSR can be defined as the extent to which the employees perceive the support extended by their employer towards themselves or other stakeholders [20,21]. Literature further classifies CSR into two subcategories: internal and external CSR. Internal CSR encompasses actions aimed at benefiting employees, whereas external CSR activities target the betterment of outsiders such as the environment, community, and customers. Despite extensive research work available in this domain, there exist some gaps which need to be addressed. First, most micro-CSR studies treat CSR perceptions as a unitary construct [17,22] and overlook the fact that employees often perceive their organizations' CSR activities in a multidimensional manner because CSR caters to diverse stakeholder groups [23]. That is, if employees/other stakeholders differentiate CSR actions of their employers according to the targeted audience, then their reactions may also vary [24,25]. Considering this, it is imperative to examine the distinct relationships of internal and external CSR perceptions with employees’ WPEB [[26], [27], [28]].

Second, literature provides contradictory findings regarding the effect of various CSR practices on employee outcomes. For example, some researchers prove the positive impact of perceived CSR on employees' attitudes and behaviors [29,30], while others prove it as insignificant [31]. Likewise, some scholars prove the direct impact of CSR on employees' WPEB [32] while others argue that CSR actions are linked with employees' WPEBs via different factors [28]. Since there exist inconsistencies in such findings, it is vital to study the complexity of this relationship [27]. Third, an increasing number of studies have focused on how various mediating mechanisms enhance this relationship. These include a green shared vision [29], CSR attributes [16], employees' empowerment and communal relationship [33], organizational commitment [17], green human resource practices [34] and so forth. However, investigation of how a green organizational climate cultivates unified green perceptions among employees for engaging in WPEB has been neglected in the literature. Since, an organization that favors a green organizational climate among employees drives these employees to participate in WPEB [13,35]; therefore, employees’ CSR perception of involvement in WPEBs can be enhanced by embedding a green organizational climate [36]. Thus, mediation of green organizational climate needs empirical examination.

Fourth, the presence/absence and strength of factors such as a green shared vision can further amplify/attenuate the involvement of employees in WPEB stemming from a green organizational climate that emphasizes green practices and conservation of resources [29]. However, the moderating role of a green shared vision in the relationship between a green organizational climate and employees' WPEB remains unclear [37]. In this regard, research highly recommends conducting further studies to examine the relevant boundary conditions in this domain and address such gaps in knowledge [34,38]. Henceforth, based on these addressed gaps, the following objectives were accomplished through an empirical investigation. First, by adopting the social exchange theory [39] and social identity theory [40] perspectives, this study examined the relationship between four distinct CSR perceptions and employees' WPEB: CSR towards the employees, community, customers, and environment. Second, this study investigates whether a green organizational climate operates as a mediator between employees' CSR perceptions and their WPEB. Finally, it discussed how a green shared vision might moderate the relationship between a green organizational climate and employees' WPEB. Consequently, this study's findings make the following substantial contributions to the literature on CSR and employees' WPEB.

1. The extensive findings can assist manufacturing firms in developing countries in understanding the significant gaps in their CSR activities and policies to not only meet their environmental obligations but also enhance their employees' environmental practices.
2. This study provides a rationale for practitioners to invest in both internal and external CSR initiatives.
3. This research will assist managers in recognizing the importance of a green shared vision among employees and the role of a green organizational climate in facilitating the implementation of green behaviors.
4. This study contributes to the expansion of the existing body of literature in the context of manufacturing industries such as those in Pakistan.

## 2 Literature review

### 2.1 Theoretical foundation

The framework of this research is grounded in the underpinnings of two theories: the social exchange theory and the social identity theory. These theories explain how internal and external CSR practices perceived by employees lead to their WPEB, the mediation of a green organizational climate in this relationship, and the moderating role of a green shared vision [41,42]. The relationship of manufacturing firms' internal CSR with employees' WPEB is explained by the social exchange theory [41]. Moreover, the relationship of organizational climate with employees' WPEB is addressed using the social identity theory [42]. First, the social exchange theory posits that when an individual receives a favor from another, there is a sense of obligation to reciprocate the favor [39]. This concept of tacit reciprocity effectively elucidates the exchange process between an organization and its employees, particularly in fostering voluntary initiatives among employees. Organizations provide employees with benefits such as fair wages, good working conditions, environmental awareness, green training opportunities for advancement, and so forth. Consequently, employees are likely to reciprocate by volunteering their time and effort to help an organization succeed [29]. According to the social exchange theory, when employees become aware of their organization's CSR activities and environmental concerns through voluntary disclosures, they engage in extra-role behavior, such as showing dedication to ecological responsibility [7]. Employees strive to align their values with the organization's pro-environmental principles, foster an organizational climate, and encourage a higher likelihood of WPEB [43].

Second, the social identity theory claims that individuals derive their identities from the workgroups to which they belong and that their affiliation with these organizations provides them with a sense of social identity [40]. As employees' perceptions of their company's internal (how they view the company) and external images (how they believe outsiders perceive the company) improve, they perceive the company as more appealing and positive. This enhanced perception fills them with a sense of pride and satisfaction [43]. Indeed, employees' assessment of their organization's image is strongly dependent on their CSR perceptions. This positive image fosters a harmonious atmosphere among employees, which synergizes with their motivation and reinforces their sense of pride in being part of the workplace [44]. Hence, employees endeavor to enhance the distinctiveness and reputation of their organization by displaying behaviors that align with their shared values [29] such as heightened commitment to voluntary participation via WPEB [40].

## 3 Hypotheses development

### 3.1 Perceived CSR towards employees

CSR towards employees refers to the policies and practices implemented in an organization that focuses on stewardship towards employees [21]. These may include good work-life balance, well-being, training and development opportunities, and diversity in the workplace [45]. Companies offering comprehensive training, competitive compensation, and effective reward systems can cultivate positive sentiments among their employees. These practices not only improve the work-related skills of employees but also empower them to contribute to their personal development, thereby generating goodwill within the organization [46]. Contrastingly, few scholars prove that CSR initiatives for employees do not always lead to the achievement of WPEB or other favorable employee outcomes. This is because employees may consider employee-centric CSR actions as part of their organization's HRM policies and an obligation towards employees rather than a favor [28]. However, conceptually CSR practices aimed at employees offer support for employees' volunteerism opportunities and benefit them beyond the legal requirements [26]. Therefore, based on mixed findings it is necessary to conduct a more thorough investigation of how an organization's CSR actions towards employees inspire employees' WPEB. That is, examining whether a green organizational climate mediates this relationship is imperative.

Through the lens of the social exchange theory, when organizations engage in employee-centric actions, they create a sense of obligation among employees to reciprocate positively, such as through WPEB [47]. When employees recognize that an organization is making intelligent and value-driven investments, they exhibit an elevated level of performance in their assigned roles. Consequently, organizations must acknowledge and incentivize these behaviors, as they not only foster a positive work environment and enhance employee satisfaction but also contribute to the overall improved organizational outcomes [46]. Such a working atmosphere creates strong commitment and is highly valued by employees, thereby resulting in the formation of a strongly embedded green organizational climate [48]. Firms that emphasize the importance of employees and treat them fairly instill a positive organizational climate [38] leading to greater involvement in WPEB [35,49]. Thus, employee-centric CSR policies implemented by organizations from a sustainable perspective tend to follow a green organizational climate. Organizational values are mirrored by employees [38]; thus, these shared values and beliefs become part of a company's culture and are embedded in a green organizational climate [50]. Hence, this study proposes the following hypothesis.

H1 *CSR towards employees has a positive relationship with a green organizational climate.*

### 3.2 Perceived CSR towards community

CSR towards the community describes the strategic efforts of firms that target the well-being of community members and society [23]. Firms that engage in activities to improve the community are recognized as role models by their employees [43]. This is because substantial participation in CSR initiatives aimed at outsiders such as community, customers or the environment are positively evaluated by outsiders [43] creating a positive social image of organizations [28]. This makes the employees feel prestigious to work with them and hence they identify themselves with such organizations [51,52]. Contrarily, literature points out that overemphasizing participation in CSR activities towards external stakeholders is considered as corporate “greenwashing” and a mere show-off for competitive advantage [53]. This can undermine organizational credibility and reduces prestige and likelihood of WPEB or other favorable employee outcomes [54]. These contrasting theoretical perspectives call for further exploration.

According to the social identity theory viewpoint, when employees identify with an organization based on its community-centric CSR initiatives, such as participation in voluntary activities, campaigns for the well-being of society, and financial support for humanitarian causes, they experience motivation and a sense of vigor [45]. When organizations adopt community-centered CSR practices, they send a powerful message to stakeholders, including employees, about their unwavering commitment to serving the community [55]. This compassion reinforces employees' identification with the organization and further enhances their motivation to attain organizational sustainability goals by engaging in WPEB [28]. Hence, organizations that prioritize CSR towards the community can create a positive work environment in which employees are not only driven by their values but also by a shared purpose to make a meaningful contribution to the community [43]. Positive perceptions of an organization's community-oriented CSR actions and the resulting efforts exerted by employees to engage in community welfare programs cultivate a caring and supportive organizational climate, known as the green organizational climate [53]. Therefore, organizational leaders focusing on sustainable societies and communities tend to incorporate a green organizational climate for their employees, representing a shared culture of environmental values and belief systems in the workplace [38]. Consequently, this study proposes the following hypothesis.

H2 *CSR towards the community has a positive relationship with a green organizational climate.*

### 3.3 Perceived CSR towards environment

In this study, perceived CSR towards the environment refers to the employee's perceptions of the adoption of eco-friendly activities by their organizations for a sustainable future. Adequate environment-centric CSR practices can provide inspiration to employees to engage in favorable behaviors [28,30], however, doubt about the true motive behind organizational CSR practices can reduce this motivation [54]. Being part of the workforce, if employees, as followers, value environment-focused CSR practices carried out by their employers, which forms an environment-sensitive ambience within the organization [36]. Specifically, in socially responsible organizations, top management places a strong emphasis on environmental concerns. This provides indispensable support and resources to employees, which assist them in amplifying the environmental performance of their organization, ultimately resulting in the cultivation of an environmentally conscious climate within the company [56].

This green organizational climate enables employees to realize the importance of green values by observing environmental rules and regulations established within the organization and nurturing WPEB [57]. The responsibility of developing a climate that encourages green tasks and interpersonal acceptance of the green contributions of employees’ rests with the leaders of organizations [36]. As these leaders are considered role models, their environment-centric CSR initiatives generate a perception among employees that the organization has a strong dedication to environmental sustainability [58]. This motivates employees to engage in green activities in workplaces that culminate in and foster a green organizational climate [36]. Firms in which grave importance is given to imparting environmentally friendly behaviors and sustainable practices [38] tend to exhibit a green organizational climate among staff [49]. Hence, this study proposes the following hypothesis.

H3 *CSR towards the environment has a positive relationship with a green organizational climate.*

### 3.4 Perceived CSR towards customers

CSR towards customers represents organizational efforts to secure consumer rights, provide full disclosure about products, implement practices that maximize customer satisfaction, and so forth [23]. When an organization proactively adopts CSR practices that prioritize benefits to customers over their interests, employees can perceive the organization as ethical and trustworthy [25,59]. Conversely, showing off motive by prioritizing customers over the employees or other stakeholders can also lessen the positive image of organizations and hence unfavorable employee outcomes [54]. Based on the social identity theory, a firm's display of benevolence towards customers provides employees with a significant and valid reason to form a strong emotional attachment and a deep sense of identification with their organization [60]. Organizations that give due consideration to customer-focused CSR activities gain respect from customers [61] and become prestigious for their employees [45]. From an employee's perspective, it is commendable for a company to prioritize customer well-being over the company's gain. Employees' trust in their organization, which stems from the organization's moral conduct through customer-centric CSR initiatives, can instill a sense of security and the belief that they are associated with a reputable and secure organization [50]. This sense of emotional attachment enables them to provide skilled professional customer service to support an organization's achievements.

It also establishes the motivation to perform job tasks to achieve organizational goals, including environment-related targets; thereby, employees opt for WPEB [28]. Hence, a shared perception of customer-oriented CSR events results in the formation of a strong green organizational climate, in which employees recognize that customer-centric CSR initiatives adopted by organizations enhance their commitment [53]. When employees collectively perceive an organization's CSR efforts towards its customers, they create a robust green organizational climate based on customer-centric values and practices. Moreover, consumers are increasingly seeking to purchase greener products as they recognize that their consumption habits have a substantial impact on the environment, which results in the adoption of green practices by organizations [62]. When organizations fulfill the requirements of environmentally sensitive customers by offering green products, it signals that employees perceive the importance of green product usage and are thus more likely to behave in an environmentally friendly way [63]. Therefore, organizations that promote environmental measures in the workplace and procure eco-friendly inputs to manufacture green products as part of their customer-focused CSR practices instigate a green organizational climate among their employees [36]. Thus, this study proposes the following hypothesis.

H4 *CSR towards the customers has a positive relationship with a green organizational climate.*

### 3.5 Green organizational climate

Over time, scholars have recognized that contextual factors have the potential to incline individuals towards adopting WPEB [64]. A green organizational climate has also emerged as a pivotal contextual factor that can shape the attitudes and behaviors of employees [65]. Organizational climate refers to employees' shared perceptions and interpretations of an organization's policies, the procedures that transform these policies into practical guidelines, and the behaviors expected and recognized by the organization [66]. This study operationalized the green organizational climate at the individual level as a unified perception among employees concerning organizational policies, practices, and procedures established to promote environmental sustainability, encompassing the range of green initiatives implemented by the organization in the workplace [65]. Predominantly, the working environment is greatly influenced by the climate maintained by organizations, leading to noticeable changes in employees' WPEB [67] which is essential for encountering ecological problems and achieving sustainability [68]. To establish common views on environmental policies among individuals, a green organizational climate provides a sustainable atmosphere among employees to activate WPEB [35,47].

According to Yeşiltaş, Gürlek and Kenar [37] environmental awareness among employees encourages the formation of a green organizational climate that manifests green behaviors in the workplace. Employees' adoption of WPEB is contingent not only on the awakening of personal environmental awareness and the systematic acquisition of environmental protection knowledge and skills but also on the presence of a positive green organizational climate [49,69]. An exemplary green organizational climate enables employees to gain a clear understanding of the organization's green values, which heightens their realization of environmental preservation and serves as a catalyst to exert additional efforts within and outside work settings [70]. Consequently, such an environment fosters an increased inclination and willingness towards WPEB. Motivated employees have high obligatory expectations for WPEB. A positive perception of outsiders contributes to the establishment of a strong organizational reputation; thus, based on the social identity theory, employees desire others to hold a favorable view of their organization [24]. A green organizational climate can help instill a positive image and internal reputation, which makes employees feel proud of their organization. Employees engage in WPEB to sustain this climate [36]. If employees perceive themselves to be immersed in a green organizational climate that fosters and encourages environmental behaviors, their propensity to engage in WPEB is heightened [35,49]. Hence, this study proposes the following hypothesis.

H5 *Green organizational climate has a positive relationship with employees' WPEB.*

### 3.6 Moderation of green shared vision

Previous research suggests the need to examine potential moderation in the relationship between green organizational climate and employees' WPEB. For instance, scholars have examined the moderating role of green values on the relationship between green organizational climate and employees' WPEB; nevertheless, they have proven that green values weaken this relationship [15]. Authors who have investigated the interaction of a shared green vision between environmental strategies and performance also recommend examining its moderation in other relationships [71]. Consequently, this study anticipates that a green shared vision will moderate the link between green organizational climate and employees' WPEB in such a way that the existence of a green shared vision will strengthen this relationship. A green shared vision refers to the collective and clear environmental strategies embraced by the members of an organization to establish environmental goals and guide strategic directions [72]. Within the context of manufacturing firms, a green shared vision has the potential to inspire employees to embrace sustainable behaviors [73]. Moreover, it can serve as a motivator for employees in manufacturing industries to strive towards an organization's sustainable development, promote green initiatives through their WPEB, excel in their environmental performance at work, and develop sustainable strategies [29]. When employees and their organizations embrace a unified vision, they are more likely to perceive their contributions as significant, leading them to feel more at ease in expressing their ideas concerning potential enhancements to the environment [74].

From the perspective of the social identity theory, comfort in expression elevates employees’ self-worth, and they identify themselves as members of a reputable organization that actively demonstrates similar concerns [29]. A green shared vision as a catalyst helps employees cultivate intrinsic motivation, thereby inspiring them to adopt green creative behavior [75] and WPEB [74]. Manufacturing organizations can utilize a green shared vision by creating awareness among employees regarding their goals and delegating responsibility for their accomplishments [73]. Similarly, Afsar et al. [74] argued that a green shared vision empowers companies to instill desired behaviors in their workforce, thus ensuring the achievement of long-term objectives. However, ineffective or no communication of goals within groups can portray the aimlessness of vision, resulting in disillusionment and distrust rather than inspiration and motivation [76]. Furthermore, employees exhibit greater involvement in environment-related activities when they perceive their organizations as socially responsible and environmentally accountable [72].

Organizational leaders foster constructive relationships in the workplace by strengthening trust among employees, offering support to team members, encouraging open communication and collaboration, and facilitating positive social interactions to assist employees in developing a constructive outlook of the environment [38]. As envisioned by top management, the collective environmental goals of the employees and their organizations result in employees' WPEB [74]. For example, training to promote energy-efficient technologies will only be fruitful if acknowledged by the members of a manufacturing organization. Therefore, it is noteworthy that a green shared vision promotes environmental knowledge sharing as well as the exchange of ideas for sustainable practices [77], especially in manufacturing firms where a green organizational climate prevails. A green shared vision encourages open dialogue, allowing employees to learn from one another and to jointly develop innovative solutions to environmental challenges. Therefore, this study proposes that by enhancing communication and collaboration, a green shared vision strengthens the relationship between a green organizational climate and the adoption of employees’ WPEB.

H6 *Green shared vision moderates the association between green organizational climate and employees' WPEB.*

### 3.7 Mediation of green organizational climate

Research indicates that employees' perceptions of CSR can encourage WPEB among them [19]. Nevertheless, the specific ways in which this happens are still not fully understood. Companies can enhance employees' trust by formulating plans and engaging in CSR initiatives that address societal issues and generate value [70]. Shiri and Jafari-Sadeghi [27] are of the view that when companies ethically address social problems, employees are more inclined to trust the company and engage in WPEB as part of a social exchange relationship. When employees perceive that their organization contributes to the greater good of society, they are more likely to share the values of their organization, fostering commitment to the organization [17]. Similarly, employees experience a heightened sense of pride when they witness their organization going beyond its CSR efforts, surpassing the actions of typical firms. This pride factor tends to get them identified by their organization's activities [78]. Thus, they return favor to their organizations by engaging in WPEB [43]. Green initiatives and norms of employees precede the environmental behavior of their leaders, because they perceive that their leaders represent themselves [11].

Leaders who prioritize environmental consciousness raise awareness among employees to protect the environment by cultivating a green organizational climate through the implementation of reward and punishment mechanisms related to environmental issues [15,69]. A green organizational climate typically emerges from behaviorally oriented actions taken by employees to protect the environment. This collective consensus motivates them to promote energy-saving behaviors within the organization [35]. Top management of socially responsible organizations places a strong emphasis on promoting WPEB by providing support and resources to employees to cultivate an environmentally conscious climate within the company [13]. In a study conducted by Zafar et al. [36] on Pakistani manufacturing companies, it was found that environmentally conscious managers are aware that employee commitment to environmental initiatives and activities in the workplace depends on a green organizational climate that maintains a positive organizational image by making employees feel proud to be part of that organization. In this line, perceived CSR actions promoting employees’ WPEB are progressively encouraged by cultivating a green organizational climate within organizations. Thus, this study proposes that.

**H**_**M1-4**_**.** G*reen organizational climate mediates between CSR towards employees, community, environment and Customers on employees' WPEB.*

**Fig. 1:**
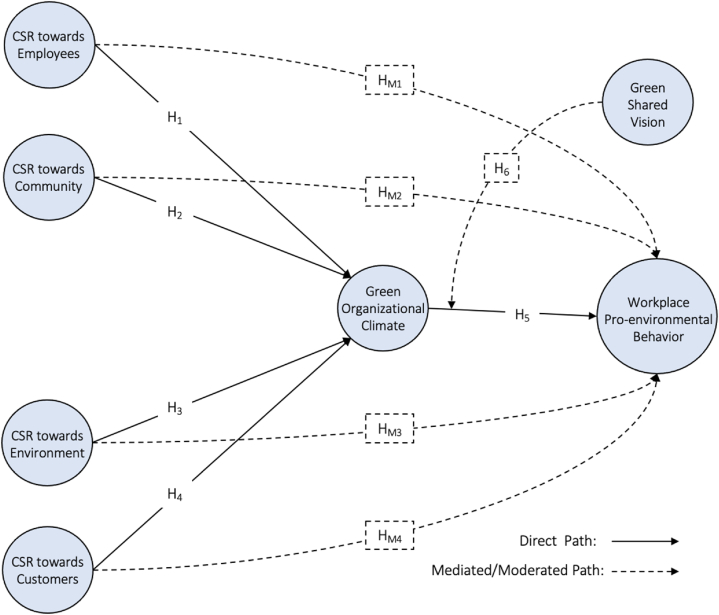
Conceptual framework.

All mediating and moderating relationships hypothesized above are presented in Fig. 1.

## 4 Methodology

### 4.1 Population and sample

The unit of analysis for this study was individuals, i.e., the population of interest comprised middle and senior management employees working in large and medium-sized manufacturing organizations in Pakistan. The manufacturing sector is responsible for causing negative environmental impacts through carbon emissions, pollutants from industrial waste, hazardous materials, depletion of natural resources, and chemical and noise pollution [1]. Particularly in developing nations such as Pakistan, harmful practices employed by manufacturing companies have led to substantial alterations in the environment [6]. Despite this, employees of manufacturing companies show greater interest in WPEB and CSR activities to engage themselves in “greening” their outlook [29]. To draw a sample of manufacturing firms in Pakistan, the Punjab province was selected, which has the largest contribution to the country's GDP generation at 54 % and employs approximately 37.6 million people [79].

Although manufacturing companies in the Punjab region are scattered across several major cities, Lahore is the largest industrial city in Punjab with the highest number of manufacturing companies [1]. The city of Lahore comprises a variety of manufacturing enterprises, including textile, metal, chemical, leather, etc., representing all the industries prevalent in other cities. The literature points out that a comprehensive sample that includes different industries would allow a deeper understanding of the correlations between the selected variables and increase the generalizability of the study [50]. Moreover, previous studies on manufacturing companies in Pakistan, such as [80], do not contain clear comparative results regarding the perceived CSR and WPEB of employees in different cities. Therefore, to improve the generalizability of the results, the data was collected from manufacturing companies in Lahore.

The sampling frame for this study was not known, as the list of medium and large manufacturing companies and the number of employees in each participating company were not available; hence, random sampling was not possible. Therefore, convenience sampling was used to identify the companies for data collection and to ensure easy access and flexibility in selecting respondents from different regions or population segments [81]. In this regard, Hair et al. [82] recommended a minimum sample size of 200 for partial least squares structural equation modeling (PLS-SEM). However, to determine an accurate minimum sample size, the G∗ Power tool suggested by Hair et al. [82] was used. By considering parameters such as effect size f^2^ = 0.15, error probability = 0.05, number of predictors = 6, and power = 0.80 [83], it determined that a minimum sample size exceeding 98 respondents was necessary. Nevertheless, to ensure robust data analysis and mitigate potential issues associated with a small sample size, this study obtained 349 valid questionnaires from the target population.

### 4.2 Data collection

The Lahore Chamber of Commerce and Industry (LCCI) maintains a comprehensive directory of companies operating in the Punjab region [1]. The R&D department of LCCI was contacted via email and asked to forward the link to the online questionnaire created in Google Forms to all organizations in their database. The questionnaire was structured and closed-ended. In particular, a screening question was added to the questionnaire to inquire about the size of each company. The definition of medium and large firms was also provided to give more clarity [84]. The questionnaires completed by employees of small companies were excluded before further screening. A total of 85 manufacturing companies responded to LCCI's official email and 58 companies agreed to participate in the study. Of these companies, 18 were clothing and textile units; 15 were food producers; 14 dealt with petrochemicals, plastic, leather, and paper manufacturers; and the remaining 11 were metal and cement manufacturers.

The data were collected from March 2023 to June 2023. A total of 370 questionnaires were received after several follow-up calls and reminders to the firms by LCCI's R&D department. During the initial screening, 21 responses were removed because of judgment errors owing to incomplete questionnaires. Of the 349 remaining useable responses, 227 were gathered from employees of medium-sized organizations and the other 122 were gathered from employees of large-sized organizations. Stringent measures were taken to maintain the confidentiality of the participants' sensitive personal and organizational information. To minimize social desirability bias, an anonymized data collection method was chosen [85]. No direct personal contact was made with organizations or their employees; all coordination took place via the LCCI platform. Furthermore, all respondents willingly and voluntarily participated in the survey.

### 4.3 Measurement items

The development of the survey questionnaire involved adapting questions that had been previously validated in a similar context. The questionnaire was administered in English, which serves as the official language for communication in Pakistan. Furthermore, the questionnaire was meticulously crafted in precise, comprehensive, and impartial language so that informants could find it engaging and express their genuine perspectives. The first part of the questionnaire asked demographic questions, including gender, age, position, education, tenure, establishment time, and firm size. The second part included items measuring employees' perceptions of various forms of CSR, green organizational climate, green shared vision, and employees' WPEB. A 5-point Likert scale was used to collect responses from the participants, where “1” denoted strong disagreement and “5” represented strong agreement. Perceived CSR was measured using 26 items based on the work of [23] with seven items for CSR towards employees, seven items for CSR towards the community, eight items for CSR towards the environment, and four items for CSR towards the customers. To assess the green organizational climate, nine items were derived from Ref. [86]. Green shared vision was measured by modifying a four-item scale from Ref. [72]. Finally, employees’ WPEB was assessed using six items from Refs. [87,88]. All items used in this study are presented in Supporting Material 1. Table S1. Survey Instrument.

### 4.4 Multivariate normality

A statistical web-based tool called “Web Power” was utilized to examine the problems associated with multivariate normality by calculating “multivariate skewness and kurtosis.” The results showed that Mardia's coefficient of multivariate kurtosis was 7.760 (t = 90.508, p < 0.01) and the skewness value was 988.519 (t = 16.994, p < 0.01). These values met the recommended threshold of p < 0.05, as proposed by Ref. [89] and indicating that the dataset used in this study did not exhibit a normal distribution.

### 4.5 Data analysis methods

Considering the presence of multivariate non-normality in the dataset, the analysis in this study employed partial least squares structural equation modeling (PLS-SEM). According to Hair et al. [82], variance-based structural equation modeling is a valuable approach for exploring non-normal datasets, enabling a comprehensive understanding of the variations within the dependent variables of the structural model. PLS-SEM recognized as an exploratory multivariate technique [90], facilitates the exploration of the relationships between latent variables. It is a causal-predictive method that does not rely heavily on rigid assumptions related to goodness-of-fit criteria, thus making it well-suited for investigating complex research frameworks comprising multiple constructs [82]. In this study, an exploratory approach was employed, integrating a multitude of exogenous factors to thoroughly examine the intricate causal associations among distinct components. Thus, PLS-SEM was determined to be the most appropriate choice for data analysis. The data were processed using SmartPLS (V4.0), which proved to be particularly beneficial for efficiently managing non-standard data in academic settings, especially when working with small datasets.

## 5 Results

### 5.1 Demographic Profile of Participants

Supporting Material 1. Table S2. Demographic Profile of Participants displays profiles of the participants. Among the respondents, 81.4 % were males and 18.6 % were females. Most respondents (46.7 %) were aged 26–35 years, 28.4 % of respondents aged 36–45 years, 14 % of respondents aged 46–55 years, 7.4 % belonged to the youthful age group of 18–25 years and only 3.5 % belonged to the 56–65 years age group. In terms of organizational position, 23.2 % of the respondents held senior management posts and 76.8 % held middle management posts. The educational qualifications of the respondents indicated that half (49.6 %) were graduate degree holders, 31.8 % were postgraduate degree holders, and 18.6 % had doctorate degrees. Moreover, the participants’ job tenure confirmed that much of the workforce 45.9 % and 43.6 % had been working for last 1–5 years and 6–10 years, respectively. Most respondents were working in medium-sized firms (65 %). In terms of “firm establishment,” more than half (50.5 %) of the respondents have been working in firms that have existed for 11–15 years.

#### 5.1.1 Common method bias (CMB)

To examine the potential impact of CMB on the study, Harman's single-factor test was used following the recommendations by Ref. [91]. This test offers a systematic approach to ascertain whether CMB exerts a considerable influence on the research model. The results revealed that a single component explained only 37.049 % of the overall variance, which fell considerably below the upper limit of 50 % proposed by Ref. [92]. Consequently, the CMB does not pose a threat to the integrity of the study's dataset. Furthermore, for CMB, this study adopted a full collinearity assessment approach, as advised by Ref. [93]. Following Kock's recommendation, all constructs were evaluated using the variance inflation factor (VIF) with the maximum threshold set at 3.3. As shown in Supporting Material 1. Table S3. Full Collinearity Test, the VIF values ranged from 1.259 to 3.220, all of which were acceptable. Therefore, this study was free from common method bias.

### 5.2 Measurement model (outer model)

**Fig. 2:**
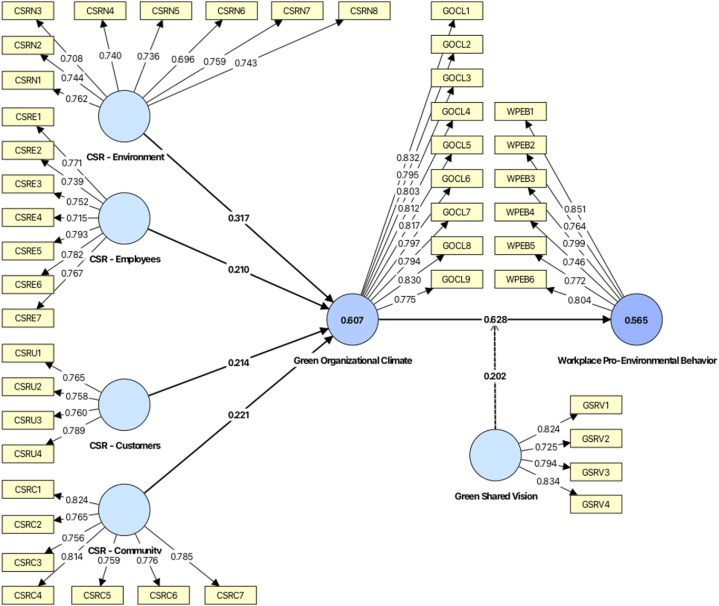
Measurement model with findings.

It is advisable to evaluate the measurement model before assessing it [82]. To guarantee the strength of the measurement model, it is essential to evaluate its internal consistency reliability, convergent validity, and discriminant validity. All analyses of the measurement model, including the corresponding values, are shown in Fig. 2.

### 5.3 Validity and reliability

The internal consistency of the constructs was assessed using statistical techniques, including Dijkstra-Hensel's rho, Cronbach's alpha, and composite reliability. The results were interpreted based on a threshold value of 0.70; values above this threshold indicated strong levels of both internal consistency and reliability [94]. As shown in Table 1, the high values of Cronbach's alpha coefficients (0.769 ≤ α ≤ 0.933), Dijkstra–Henseler's Rho-A coefficients (0.771 ≤ RhoA ≤0.933), and composite reliability (0.852 ≤ CR ≤ 0.944) indicate that all measures used in this research are highly reliable. Convergent validity was analyzed using the average variance extracted (AVE) method, which estimates the proportion of variance associated with latent constructs [82]. The AVE values (0.578 ≤ AVE ≤0.650) are higher than the cutoff value of 0.5 [82], implying that the measures are valid.

**Table 1:** Validity and reliability.

Variables	No. Items	Mean	Standard Deviation	Cronbach's Alpha	Composite reliability (rho_a)	Composite reliability (rho_c)	Average Variance Extracted	Variance Inflation Factors
CSRE	7	2.445	0.898	0.878	0.880	0.905	0.578	2.640
CSRC	7	2.358	0.939	0.895	0.897	0.917	0.613	2.533
CSRN	8	2.279	0.788	0.879	0.880	0.905	0.542	2.245
CSRU	4	3.018	0.952	0.769	0.771	0.852	0.590	1.206
GOCL	9	3.111	0.945	0.933	0.933	0.944	0.650	1.082
GRSV	4	3.237	0.984	0.805	0.812	0.873	0.632	1.056
WPEB	6	3.558	0.935	0.879	0.881	0.909	0.624	–

**Note:** CSR towards employees (CSRE), CSR towards community (CSRC), CSR towards environment (CSRN), CSR towards customers (CSRU), Green organizational climate (GOCL), Green shared vision (GRSV), Workplace pro-environmental behavior (WPEB).

### 5.4 Discriminant validity

Discriminant validity is commonly assessed through cross-loadings, the heterotrait-monotrait (HTMT) ratio, and the Fornell-Larcker criterion. The Fornell-Larcker criterion determines whether a construct fulfills discriminant validity by comparing the square root of its average variance extracted (AVE) with the square roots of the AVEs of other latent variables in the same row and column [82]. In Supporting *Material 1*. Table S4*. Fornell-Larcker criterion*, the Fornell-Larcker criterion values for all the constructs are highlighted in bold. These values (ranging from 0.736 to 0.806) are higher than any correlations within the respective rows and columns containing these constructs, indicating satisfactory discriminant validity. Moreover, to establish significant discriminant validity, all HTMT values must remain below the specified threshold of 0.90 [95]. As illustrated in Supporting *Material 1.* Table S5*. Heterotrait-monotrait ratio (HTMT) - Matrix*, the HTMT values for all components were observed to be within the designated threshold, authenticating the presence of discriminant validity.

To validate the model, a cross-loading analysis was conducted to compare the outer loadings of the structures. All loadings should exceed 0.60 to establish strong model validity [90]. Supporting Material 1. Table S6. Loading and Cross loadings highlight the construct factor loadings in bold italic font and reveal that all loadings (ranging from 0.696 to 0.851) surpassed the suggested threshold. Overall, by subjecting the factors to all three types of validity tests, this study confirmed the presence of strong discriminant validity.

### 5.5 Structural model (inner model)

As suggested by Hair et al. [82], a structural model assessment should focus on multicollinearity issues, path coefficients, explanatory power, and predictive power. To address multicollinearity, it is important to ensure that the VIF for each construct does not exceed three [94]. The data presented in Table 1 shows that none of the VIF values exceeded the specified threshold, confirming that there was no multicollinearity among the constructs.

### 5.6 Hypotheses testing

This study used 5000 bootstrapping to analyze the inner structural model. Results given in Table 2 reveal that CSR towards employees has a significant positive relationship with green organizational climate (β-value = 0.210, t-value = 3.289, and p-value <0.001), which confirms H1. Similarly, CSR towards the community positively relates to the formation of a green organizational climate (β-value = 0.221, t-value = 3.413, and p-value <0.001), supporting H2. CSR towards the environment exhibits a significantly positive relationship with green organizational climate, with a β-value of 0.317, t-value of 5.679, and p-value <0.001; this proves H3. Likewise, CSR towards the customers has a strong relationship with green organizational climate (β-value = 0.214, t-value = 4.991, and p-value <0.001), verifying H4. Furthermore, the green organizational climate has a significant positive relationship with employees’ WPEB (β-value of 0.628, t-value of 18.291, and p-value <0.001). This strongly supports H5. An examination of the confidence intervals (CI-Min and CI-Max) for all correlations indicated that the value zero (0) did not fall within these ranges. This finding provides additional support for the associated hypotheses and reinforces the strength and reliability of our results [82].

**Table 2:** Hypotheses testing.

Hypotheses		Beta	CI Min	CI Max	*t* Value	*P* Value	*f*^*2*^	*R*^*2*^	Decision
H_1_	CSRE → GOCL	0.210	0.107	0.316	3.289	0.001	0.043	0.607	Supported
H_2_	CSRC → GOCL	0.221	0.112	0.326	3.413	0.000	0.049		Supported
H_3_	CSRN → GOCL	0.317	0.227	0.410	5.679	0.000	0.114		Supported
H_4_	CSRU → GOCL	0.214	0.145	0.286	4.991	0.000	0.096		Supported
H_5_	GOCL → WPEB	0.628	0.571	0.683	18.291	0.000	0.839	0.565	Supported
*Moderation of Green Shared Vision*									
H_6_	GOCL∗GRSV→ WPEB	0.291	0.215	0.370	6.155	0.000	0.099		Supported
*Mediation of Green Organizational Climate* (H_M1-4_)									
H_M1_ CSRE → GOCL → WPEB		0.132	0.066	0.204	3.123	0.001			Supported
H_M2_ CSRC → GOCL → WPEB		0.139	0.071	0.204	3.417	0.000			Supported
H_M3_ CSRN → GOCL → WPEB		0.199	0.140	0.262	5.368	0.000			Supported
H_M4_ CSRU → GOCL → WPEB		0.134	0.092	0.180	4.956	0.000			Supported

**Note:** CSR towards employees (CSRE), CSR towards community (CSRC), CSR towards environment (CSRN), CSR towards customers (CSRU), Green organizational climate (GOCL), Green shared vision (GRSV), Workplace pro-environmental behavior (WPEB).

### 5.7 Mediation analysis

Table 2 also illustrates the mediation of green organizational climate between various forms of perceived CSR and employees' WPEB. This confirms that the results for all four associations; CSR towards employees to their WPEB, CSR towards the community to employees' WPEB, CSR towards the environment to employees' WPEB, and CSR towards customers to employees’ WPEB were mediated by a green organizational climate. Moreover, the CI Min and CI Max values did not contain zeros (0) in the intervals. These results indicate the full mediation of the green organizational climate, which supports H_M1_–H_M4._

### 5.8 Moderation analysis

Table 2 further pinpoints that a green shared vision significantly moderates the correlation between a green organizational climate and employees’ WPEB. This is evident from the statistical measures (β-value of 0.291, t-value of 6.155, and p-value <0.001). Also, an examination of the minimum and maximum confidence intervals (0.215 and 0.370) shows that the range does not encompass the value 0. Thus, these results support H6.

### 5.9 The coefficient of determination (R^2^)

R^2^ determines the proportion of variance in the dependent variable that can be explained by the independent variable(s). According to Hair et al. [94], substantial, moderate, and weak endogenous latent variables are associated with R^2^ values of 0.75, 0.50, and 0.25, respectively. Table 2 provides an overview of the five exogenous constructs in the model. The green organizational climate has an R^2^ value of 0.607, indicating that the exogenous factors related to it can explain 60.7 % of the variation, while the R^2^ value for employees’ WPEB is 0.565, representing 56.5 % of the variation explained by green organizational climate. Both values suggested moderate explanatory power based on the exogenous factors included in the model [94].

### 5.10 The effect size (f^2^)

F^2^ determines the substantial relationship between exogenous factors on endogenous variables. Unlike measuring shared variation, f^2^ focuses on the individual variances of each variable [82]. As defined by Cohen [96], f^2^ is categorized based on the magnitude of its effects into three ranges: small (f^2^ ≥ 0.02), medium (f^2^ ≥ 0.15), and large (f^2^ ≥ 0.35). The values corresponding to f^2^ are listed in Table 2. These values indicate that only a green organizational climate (0.839) had a large effect on employees' WPEB. The analysis further revealed small effect sizes for employee-centric CSR on the green organizational climate (f^2^ = 0.043), community-centric CSR on the green organizational climate (f^2^ = 0.049), environment-focused CSR on the green organizational climate (f^2^ = 0.114), customer-focused CSR on the green organizational climate (f^2^ = 0.096), and green shared vision on employees’ WPEB (f^2^ = 0.099).

### 5.11 PLS predict *(Q*^*2*^*)*

Researchers using PLS-SEM and regression-based methods have often overlooked the evaluation of a model's ability to predict outcomes beyond the observed data. Instead, they primarily rely on the R^2^ measure, which solely reflects the model's explanatory capacity and does not provide any indication of the model's effectiveness in predicting outcomes beyond the data [97]. Therefore, to validate the explanatory power of the sample, this study employed out-of-sample predictive relevance via PLS_predict_ analysis [98]. The findings in Supporting Material 1. Table S7. PLS Predict shows that the *Q*^*2*^_predict_ values are greater than zero, indicating the model's ability to predict outcomes beyond the observed data. Additionally, the root mean squared error (RMSE) values of the PLS-SEM and the linear regression model (LM) were compared. All RMSE-PLS values were lower than the RMSE-LM values, suggesting a high level of predictive power [94] for both endogenous constructs green organizational climate and employees' WPEB.

### 5.12 Multi-group analysis

To examine the variance and statistically significant differences in parameter estimation between the study groups (i.e., samples from medium and large firms), this study employed nonparametric methods [82] within the framework of Partial Least Squares Multi-Group analysis (PLS-MGA). However, it is essential to establish measurement invariance. The measurement invariance of the composite (MICOM) method was used, which involves step-by-step fulfillment of configural invariance, compositional invariance assessment, and equal means and variances [99]. Supporting Material 1. Table S8. MICOM Analysis shows that the permutation p-values for all constructs are larger than 0.05, which establishes measurement invariance, indicating that this study can continue to compare the differences between medium and large firms through the MGA.

Table 3 further presents PLS-MGA results after the successful fulfillment of MICOM. All group comparisons for H1, H2, H3, H5, and H6 were insignificant as p-values >0.05, except for H4 where p-value = 0.021. Therefore, there exists a notable variation between large and medium manufacturing firms in Pakistan in terms of the relationship between CSR towards customers and a green organizational climate.

**Table 3:** Multi-group analysis.

Hypothesis		Original (Large)	Original (Medium)	Difference (Medium - Large)	2-tailed (Medium vs Large) p value
H_1_	CSRE → GOCL	0.225	0.205	−0.020	0.448
H_2_	CSRC → GOCL	0.243	0.202	−0.041	0.375
H_3_	CSRN → GOCL	0.377	0.274	−0.102	0.188
H_4_	CSRU → GOCL	0.110	0.283	**0.173**	**0.021**
H_5_	GOCL → WPEB	0.595	0.640	0.044	0.277
H_6_	GOCL∗GRSV→ WPEB	0.129	0.231	0.102	0.119

**Note:** CSR towards employees (CSRE), CSR towards community (CSRC), CSR towards environment (CSRN), CSR towards customers (CSRU), Green organizational climate (GOCL), Green shared vision (GRSV), Workplace pro-environmental behavior (WPEB).

## 6 Discussion

Numerous scholars have studied the relationship between perceived internal [100], perceived external CSR [29,62,100,101], and both perceived internal and external CSR with employees' WPEB [16,102]. This study aimed to understand how a green organizational climate mediates the relationship between both (internal and external) CSR perceptions of employees and their WPEB. Additionally, we investigated the moderating role of green shared vision in the association between employees’ CSR perceptions and their WPEB, particularly within the context of the manufacturing sector in Pakistan. Potential explanations and interpretations are presented after elucidating the results in the context of earlier research findings. The demographic profiles of the respondents show that the gender distribution in the data set is unequal, with a higher number of male respondents than female respondents. However, the ratio of males to females in the dataset accurately reflects the gender distribution in the manufacturing sector of Pakistan. Gender-specific employment statistics show that only 14 % of women are employed in the manufacturing sector, while the rest are employed in the service sector and agriculture. A smaller number of women (12 %) are economically active in urban areas such as Lahore. Furthermore, the labor force in Pakistan is dominated by men, 84 % of whom are employed, while the female labor force participation rate is only 26 % [103]. The demographic data also shows that most of the participants in this study belong to the young and middle age groups. One reason for this trend is that the targeted sample only included middle and senior management employees. Top management such as Chief Executive Officers who formulate CSR policy, lower management and non-executive employees were not included in the sampling; therefore, the participation rate of youth and respondents above 56 years of age was only 7.4 % and 3.5 % respectively. These demographic characteristics are in line with previous studies such as that of [1] conducted in the context of manufacturing companies in Pakistan.

Furthermore, based on empirical results of hypotheses testing, this study demonstrates a significant relationship between perceived CSR and employees' WPEB. This finding is in line with recent study by Cheema, Afsar and Javed [50], who used a cross-industry sample. These results emphasize that firms must realize the imperativeness of CSR towards employees to stimulate their WPEB; in other words, by prioritizing measures beneficial for employees, such as work-life balance, organizations can create a sense of happiness in their workforce, leading to greater job satisfaction and motivation to reciprocate. This, in turn, encourages employees to go beyond their prescribed duties and engage in additional roles such as WPEB. Subsequently, the study revealed that CSR for the community had a significant relationship with employees' WPEB. This finding aligns with similar studies by Shiri and Jafari-Sadeghi [27] on Iranian food businesses, and Luu [104] on the Vietnamese hotel industry. An explanation could be that employees feel prestigious about being associated with an organization that actively contributes to the community's well-being. This sense of organizational pride [43] can motivate employees to align their behavior with the organization's values, including environmental ones.

Moreover, employees recognize that their organization's efforts are part of a broader societal impact and feel a greater obligation for philanthropic aspects. This heightened sense of social responsibility can drive employees to actively engage in WPEB. The results also revealed that environmental CSR has a significantly positive relationship with employees' WPEB. Previous studies conducted in several countries found similar associations [29,101]. The results indicate that environmental CSR triggers employees' WPEB in Pakistani manufacturing firms by inculcating a feeling of social duty among employees and serving as a motivating factor for adopting and implementing practices aimed at environmental protection. Hence, through sound CSR practices, employees achieve environmental knowledge, awareness, and prestige, thus engaging in sustainable behaviors and contributing to a greener and more socially responsible workplace.

Next, this study proves that CSR for customers has a positive relationship with employees' WPEB. This outcome aligns with the previous investigations by Suganthi [62], and Afsar and Umrani [105]. A reasonable explanation is that manufacturing firms in Pakistan involve employees in responsible product development and CSR initiatives to benefit customers. When employees actively participate in the development of products that prioritize customer satisfaction by offering sustainable products at reduced costs, they tend to develop a strong awareness and sense of responsibility towards environmental sustainability. Employees’ involvement in sustainable product manufacturing reinforces their understanding of the importance of eco-friendly outputs, which leads to WPEB. Moreover, when organizations give due diligence to consumer rights, customers give positive feedback and spell favorable word-of-mouth about the organization, which in turn becomes a matter of pride for employees [43]. Employees become emotionally attached to the organization and exhibit a sense of ownership in their workplace, leading to their WPEB such as reduced energy consumption and advocating for sustainable practices within the organization.

In particular, the MGA shows that there exists a sizeable difference in the relationship of CSR towards customers with green organizational climate across medium-sized and large-sized firms, such that the medium-sized firms provide greater opportunities to foster green organizational climate through CSR towards customers as compared to large-sized firms. Some plausible causes for this dissimilarity include the greater involvement of medium-sized firms in CSR towards customer practices, their close link with customers due to smaller-scale operations, a more localized approach, greater access to niche environment-sensitive customers, and comparatively fewer resource constraints due to their less complex structure and streamlined decision-making. Furthermore, the findings confirmed that a green organizational climate significantly enhances employees’ WPEB. This association is consistent with the findings of Zafar et al. [36] for the Pakistani textile industry, and Iqbal and Piwowar-Sulej [38] for the Chinese manufacturing enterprises. These findings indicate that when organizations prefer and encourage sustainable practices, they create an environment that encourages employees to engage in WPEB. This includes initiatives such as waste reduction, energy conservation, and the use of eco-friendly technologies and materials.

Moreover, when organizations appreciate employees' WPEB through incentives, rewards, and recognition programs, it further enhances their commitment to sustainable action [6]. As a result, a strong green organizational climate positively improves employees' WPEB, as in Pakistani manufacturing firms. This study provides additional empirical evidence to identify the mediation of green organizational climate between perceived CSR types and employees' WPEB. Although CSR participation has become a sustainable corporate policy for many organizations, and its importance in deriving employees' WPEB is recognized; however, when and how employees' perceptions of CSR activities lead to their WPEB is less understood [16,29]. To fill this gap, this study considers the mediation of a green organizational climate. It is plausible to expect the top management to induce a culture in organizations that can create a system of beliefs and norms within the organization via a green organizational climate to help employees promote WPEB [36]. Leaders can act as role models for employees by showing greater concern for employees’ WPEB and understanding that this behavior is only possible if a green organizational climate is implemented in a true spirit [38].

As such, when employees' values and beliefs regarding the environment strongly match those of their organizations, a green organizational climate develops through which employees' favorable CSR perceptions of their organizations inspire them to practice WPEB [50]. Likewise, a noteworthy finding of this study is that a green shared vision significantly moderates the relationship between employees' perceived CSR and their WPEB. This implies that employees with a higher level of green shared vision participate more in WPEB than employees with a low green shared vision. This result aligns with the research of Zhao et al. [75], who demonstrated that the inclusion of a green shared vision enhances the green creative behavior of employees, while its absence can lower it. Thus, it is inferred that manufacturing firms in Pakistan must boost the green shared vision among employees so that they can reap greater benefits in terms of employees' WPEB. In an organization where an eco-friendly culture is promoted and rewarded, employees’ motivation to exercise WPEB increases. It provides them with an opportunity to align their goals with the collective environmental mission of the organization. By embracing this unified vision, employees can align their environmental concerns and behaviors with those of the organization.

## 7 Conclusion

Under the premise of social exchange and social identity theories, this study examined the relationship between employees' CSR perceptions and their WPEB in the context of manufacturing companies in a developing country, Pakistan. The study further examined the mediating role of green organizational climate in enhancing the relationship between various perceived CSR practices and employees' WPEB. The study also investigated the moderating role of green shared vision in the above-examined relationships. From the results, it was concluded that employees' perceptions of all four types of organizational CSR initiatives namely CSR towards employees, customers, community and the environment significantly relate to their tendency to engage in WPEB. Nevertheless, perceived CSR towards the environment contributed the most to employees' inclination towards WPEB. Findings further revealed that when organizations adopted and maintained a green organizational climate, it significantly helped employees get inspiration from the organization's CSR actions and transform them into their own WPEB. Another important finding of this research pinpointed the mediated moderation relationship of green shared vision and organizational climate. It confirmed that when employees across various levels of the organization shared the same viewpoint in favor of green initiatives, the benefit of inducing a green climate was amplified, and so did its mediating role between perceived CSR and employees' WPEB.

### 7.1 Theoretical implications

The results of this study provide crucial theoretical insights that enrich the existing literature on perceived CSR and environmental management. This study contributes to the existing body of knowledge in several ways. First, it fulfils the call of Raza et al. [43] and Ahmed et al. [106] for further research that considers different CSR dimensions as antecedents and investigates which dimension is more important for employees to improve their WPEB. Therefore, this study is a pioneer in proposing and separately analyzing all four dimensions of perceived CSR in a framework. However, previous researchers have primarily considered one dimension at a time [29], or combine all dimensions into a single construct [43,62,105,106]. This study's framework addresses this important gap in the literature by integrating both internal and external perceived CSR among the employees of manufacturing firms in Pakistan. Second, this study incorporates two prominent theories (social exchange theory and social identity theory) to illustrate how employees' WPEB can be enhanced by firms' CSR initiatives, a green organizational climate, and a green shared vision. The relationship of a company's internal CSR is explained using social exchange theory, and the relationship of external CSR (which focuses on the community, customers and the environment) is explained using social identity theory. Third, this study extends the understanding that a green organizational climate plays a pivotal role as a mediator in enhancing the relationship between perceived CSR towards employees, the community, the environment, and customers and employees' WPEB, which has not been extensively studied.

Fourth, this study not only investigated the underlying mechanism linking perceived CSR to employees' WPEB but also established the boundary condition of a green shared vision that reinforced the relationship between green organizational climate and employees' WPEB. This moderation of green shared vision was rarely explained in previous studies. To address this gap in the literature, this study clarifies how a strong and weak green shared vision can strengthen/weaken the execution of employees' WPEB. This finding is paramount because the indirect relationship between organizational CSR activities and employees' WPEB depends on employees' green shared vision. This means that the more an organization's employees share the same vision regarding environmental protection and preservation, the more they will show a collective concern for the betterment of the environment, and vice versa. Next, previous scholars have not given much consideration to examining whether CSR perceptions have a different relationship with employees' WPEB across firms of distinct sizes. To address this gap, the MGA performed in this study highlights that, unlike large manufacturing firms in Pakistan, customer-centric CSR initiatives by medium-sized firms have a greater propensity to develop a green organizational climate. Overall, this study significantly adds to the relevant literature by providing a comprehensive analysis of the factors which significantly relate to employees' WPEB at both the individual and organizational levels. Specifically, by examining the moderation of green shared vision on green organizational climate and employees' WPEB link, this study offers a profound insight into the intricate nature of this relationship.

### 7.2 Practical implications

This study has several important practical implications. First, the results indicate that a positive perception of a company's CSR activities towards all key stakeholders, such as employees, customers, the community and the environment, leads to a positive WPEB of employees. Therefore, the top management must recognize the importance of investing in both internal and external CSR initiatives to cultivate and maintain a culture of eco-friendly conduct among employees. One way to achieve this is that companies proactively communicate their external CSR activities such as environmental efforts, social welfare initiatives, and customer-centric programs to their workforce. Second, organizations must make their employees realize that they are being given benefits beyond the legal requirements and that they are important to the employers. For example, businesses can convey internal and external CSR actions by implementing in-house communication strategies. Moreover, they should ensure that their employees have easy access to comprehensive information on the significance, diversity, and value of their CSR initiatives.

Managers can play a crucial role in translating environmental orientations into actionable practices. They can serve as role models for implementing WPEB by establishing a climate that promotes eco-consciousness, encourages and supports green initiatives from staff, and facilitates employees’ engagement in WPEB. By doing so, managers can inspire and motivate other employees to become environmentally conscious of their workplace actions. Thus, it is central for organizations, particularly manufacturing firms, to consistently impart organizational efforts towards various CSR initiatives and environmental policies to their employees to not only foster a positive perception but also enhance their perceptions regarding CSR. Employees often remain insufficiently informed about the strategies that the organization opts to address social responsibility issues. Despite sincere investments and genuine attempts by organizations to make positive contributions to all stakeholders, employees may harbor skepticism and doubt about CSR activities because of insufficient knowledge. To proactively achieve this objective, organizations should utilize various communication channels such as newsletters, magazines, and popular social media platforms to advertise their CSR work by targeting a diverse group of stakeholders.

Furthermore, this study highlights the significance of enhancing the green organizational climate within an organization through CSR initiatives to enable employees' WPEB. In addition, when a company genuinely integrates CSR principles into its operations, it leads to a more cohesive and shared perception of green policies and procedures among its employees. Consequently, organizations must authentically incorporate CSR into their practices and align them with their mission and vision statements. Organizations should also emphasize environmental education and training aimed at ensuring WPEB among their employees. Training programs play a vital role in instilling a sense of social responsibility in the workforce and serve as a prominent means of achieving this purpose. Most importantly, the variation across large and medium-sized organizations regarding the relationship between CSR practices towards customers and a green organizational climate indicates that larger firms could further enhance their customer-oriented CSR actions to match those of medium-sized firms. This can be regulated by improving their participation in customer-oriented CSR initiatives, building closer relationships with customers, and identifying the barriers that cause this variation. This research pinpoints another important aspect regarding female labor force participation in Pakistan's manufacturing sector. As employment in this sector is dominated by men, companies should also promote women through equal opportunities in employment. Specifically, in line with the UN's SDGs such as goal number 5 (gender equality), organizations should ensure that during their hiring and promotion process, full consideration is given to female employees to participate in the decision-making process and hold a sizeable number of managerial positions.

### 7.3 Policy implications

This study also offers several policy implications which are applicable to enterprises in both the developed and developing countries. First, when the government encourages WPEB among the employees of enterprises, it should implement strict CSR regulations for these enterprises. Government should also focus on creating and boosting environmental climate within these firms through which greater developmental benefits can be reaped in providing necessary inspiration to employees via CSR. For instance, in 2019, the Pakistani government executed CSR regulations and held the manufacturing firms accountable for unsustainable practices [29]. Secondly, the government can establish a platform for organizations to organize experience-sharing sessions on creating an atmosphere to promote WPEB among the employees and importance of CSR in building employees’ motivation towards WPEB. This can offer learning opportunities for other organizations. Third, the government can also consider introducing CSR excellence awards to promote exemplary CSR practices. Such recognition would be an incentive for enterprises to implement the most commendable CSR initiatives. The prospect of receiving these awards could encourage companies to take serious steps to reduce the adverse environmental impacts of their operations. Consequently, this could lead to increased participation of enterprises in CSR activities as well as enhanced WPEB by their employees. Fourth, apart from the role of organizations in introducing environmental training programs for their employees, the government and policymakers should also provide their support for such awareness and training programs. Particularly, government agencies and chambers of commerce should plan detailed and funded environmental knowledge certification programs for organizations and their employees. Such sponsored programs would not only increase the interest of organizations and their employees in certified environmental training but would also motivate them to show progress after the training and apply appropriate incentives.

## Limitations and directions for future research

In addition to offering significant contributions to theory and practice, this study acknowledges certain limitations that present promising avenues for future research. First, although the findings of current study are applicable within the context of manufacturing organizations in other developing countries and emerging economies yet extending them beyond a single sector and country is worth consideration. For instance, exploring similar phenomena in a country comparative study (such as between developed and developing countries) can enhance the robustness of the results. Secondly, a cross-sectional approach was employed to gather data on the study variables. Since causal relationships cannot be estimated using cross-sectional data, a longitudinal study with an experimental design is recommended for future investigations. Thirdly, it is important to acknowledge that the data in this study was solely collected from middle and top managers, with the perspective of operational staff being excluded. However, the operational staff directly involved in manufacturing can provide valuable and unique insights from an operational perspective. Therefore, future researchers should consider incorporating the viewpoints of operational staff to enrich the findings and implications.

Next, this study included a green organizational climate as a mediating variable. Future studies could include other variables such as employees’ goal orientation, job satisfaction, and green crafting to investigate their possible mediating roles. Fifth, the moderator, green shared vision, measures the unified perception of environmental sustainability among employees of an organization. Future scholars should consider other negative and positive perceptions as moderating factors such as CSR skepticism, CSR authenticity, and perceived greenwashing. Future scholars could also examine the complementarity relationship of internal and external CSR perceptions with individual-level employee outcomes. Sixth, to overcome social desirability bias, this study employed an anonymized data collection approach, where researchers and participants were not in any direct contact, thus minimizing any chances of dishonest responses. However, in future research, specific questions addressing social desirability bias may be added to the questionnaire for further mitigation of this issue.

Seventh, this study opted for data collection from only Lahore. This approach was suitable because Lahore is considered the industrial hub of Punjab province in Pakistan and has a maximum concentration of manufacturing firms. However, future research should consider taking samples of manufacturing firms from other cities as well. This could help in a detailed understanding of Pakistan's manufacturing sector and enable comparisons of CSR participation and execution of employees' WPEBs between the manufacturing firms in different areas of Pakistan. Future research should also broaden the focus beyond the manufacturing industry and include different sectors, such as banking and hospitality, to achieve more comprehensive and generalizable results.

Another limitation of this research is the gender distribution in the dataset. Although the study provided reasonable grounds for this variation within the manufacturing sector's context, it must be carefully considered in future research. Finally, this study adopted a quantitative approach. However, to gain a deeper understanding of the relationship between CSR and employees' WPEB, future studies should employ qualitative or mixed methods approaches.

## Ethical approval

The human research ethics committee of University of Engineering and Technology, Lahore has approved this study (Approval Number: Uni/IB&M/Dir/2023).

## Consent to participate

Written informed consent was obtained from respondents who participated in the survey.

## Consent to publish

All authors approved the manuscript and gave their consent for submission and publication.

## Funding

This research received no specific grant from any funding agency in the public, commercial, or not-for-profit sectors.

## Availability of Data and materials

The original contributions presented in the study are included in the article/Supplementary Material (S2. Dataset), further inquiries can be directed to the corresponding author/s.

## CRediT authorship contribution statement

**Sara Kanwal:** Writing – original draft, Methodology, Investigation, Conceptualization. **Abdullah Al Mamun:** Writing – review & editing, Methodology, Formal analysis, Conceptualization. **Mengling Wu:** Writing – original draft, Methodology, Investigation, Conceptualization. **Saad Mahmood Bhatti:** Writing – original draft, Methodology, Investigation, Conceptualization. **Mohd Helmi Ali:** Writing – review & editing, Methodology, Formal analysis, Conceptualization.

## Declaration of competing interest

The authors declare the following financial interests/personal relationships which may be considered as potential competing interests: Corresponding author is the associate editor of Heliyon (Business and Management) If there are other authors, they declare that they have no known competing financial interests or personal relationships that could have appeared to influence the work reported in this paper.
