# Supplementary material for: Corporate social responsibility: A Driver for green organizational climate and workplace pro-environmental behavior
Source: Heliyon. 2024 Oct 5;10(19):e38987. doi: 10.1016/j.heliyon.2024.e38987 (PMC11490784; doi:10.1016/j.heliyon.2024.e38987)
Supplement: Multimedia component 2 [file mmc2.docx]

**Table S1.** Survey Instrument

| Code | Items |
| --- | --- |
| CSRE1 | Our organisation supports equal opportunities at work (example: gender equality policies). |
| CSRE2 | Our organisation encourages employees' diversity in the workplace. |
| CSRE3 | Our organisation avoids all forms of discrimination in its recruitment and promotion policies. |
| CSRE4 | Our organisation supports employees who want to acquire additional education. |
| CSRE5 | Our company's policies encourage employees to advance in their careers and skills. |
| CSRE6 | Our organisation implements flexible policies to provide a good work-life balance for its employees. |
| CSRE7 | The managerial decisions related to the employees are usually fair. |
| CSRC1 | Our organisation contributes to improving the well-being of populations by providing help for schools, sporting events, and so on. |
| CSRC2 | Our organisation invests in the health of the population (e.g., vaccination, fight against AIDS). |
| CSRC3 | Our business supports local sports and cultural activities. |
| CSRC4 | Our organisation provides financial support for humanitarian causes and charities. |
| CSRC5 | Our organisation contributes to the campaigns and projects that promote the well-being of society. |
| CSRC6 | Our organisation assists nongovernmental organisations working in troubled areas. |
| CSRC7 | Our organisation encourages its employees to participate in voluntary activities. |
| CSRN1 | Our organisation targets sustainable growth, which considers future generations. |
| CSRN2 | Our organisation encourages its members to adopt eco-friendly behaviors (sort trash, save water, and use less energy). |
| CSRN3 | Our organisation acts to reduce pollution related to its activities (e.g., choice of materials, eco-design, |
| CSRN4 | Our organisation contributes toward saving resources and energy (e.g., recycling and waste management). |
| CSRN5 | Our organisation respects and promotes the protection of biodiversity. |
| CSRN6 | Our organisation invests in clean technologies and renewable energies. |
| CSRN7 | Our organisation implements special programs to measure and minimize its negative impact on the natural environment. |
| CSRN8 | Our organisation participates in activities that aim to protect and improve the quality of the natural environment. |
| CSRU1 | Our organisation has an effective consumer grievance handling mechanism. |
| CSRU2 | Customer satisfaction is highly important for our organization. |
| CSRU3 | Our organisation provides full and accurate information about its products to its customers. |
| CSRU4 | Our organisation protects consumer rights beyond the legal requirements. |
| GOCL1 | Our organisation often publicises information about environmental protection measures. |
| GOCL2 | Our organisation stresses observing environmental regulations and laws. |
| GOCL3 | Our organisation continues to provide employees with environmental education and training. |
| GOCL4 | Our organisation participates in local or community environmental activities. |
| GOCL5 | Our organisation promotes environmental measures in the workplace. |
| GOCL6 | Our organisation requests that employees consider environmentally friendly products when making purchase decisions. |
| GOCL7 | Our organisation emphasises energy-saving policies and measures. |
| GOCL8 | Our organisation demands waste volume reduction. |
| GOCL9 | Our organisation stresses resource recycling. |
| GRSV1 | There is a commonality of environmental goals in our organization. |
| GRSV2 | There is total agreement on our organization's strategic environmental direction. |
| GRSV3 | All members in our organization are committed to the environmental strategies of the organization. |
| GRSV4 | Our organization's employees are enthusiastic about the collective environmental mission of the organization. |
| WPEB1 | In an effort to increase my organization's environmental performance, I make suggestions and bring new ideas about environmentally friendly practices to environmental committees. |
| WPEB2 | I fulfil the responsibilities specified in my job description in environmentally friendly ways. |
| WPEB3 | At work, I recycle (e.g., paper, cans, batteries, and oil). |
| WPEB4 | When I leave the office, I turn off the lights. |
| WPEB5 | At work, I take part in environmentally friendly programs. |
| WPEB6 | I share my knowledge about the environment with co-workers. |

**Note:** CSR towards employees (CSRE), CSR towards community (CSRC), CSR towards environment (CSRN), CSR towards customers (CSRU), Green organizational climate (GOCL), Green shared vision (GRSV), Workplace pro-environmental behavior (WPEB)

**Table S2.** Demographic Profile of Participants

|  | N | % |  |  | N | % |
| --- | --- | --- | --- | --- | --- | --- |
| *Gender* |  |  |  | *Education* |  |  |
| Male | 284 | 81.4 |  | Bachelor’s or equivalent | 173 | 49.6 |
| Female | 65 | 18.6 |  | Master’s degree or equivalent | 111 | 31.8 |
| Total | 349 | 100.0 |  | Doctoral degree or equivalent | 65 | 18.6 |
|  |  |  |  | Total | 349 | 100.0 |
| *Age Group* |  |  |  |  |  |  |
| 18–25 years | 26 | 7.4 |  | *Tenure* | | |
| 26–35 years | 163 | 46.7 |  | Less than 1 year | 12 | 3.4 |
| 36–45 years | 99 | 28.4 |  | 1–5 years | 160 | 45.9 |
| 46–55 years | 49 | 14.0 |  | 6–10 years | 152 | 43.6 |
| 56–65 years | 12 | 3.5 |  | 11–15 years | 12 | 3.4 |
| More than 65 years | 0 | 0 |  | 16–20 years | 13 | 3.7 |
| Total | 349 | 100.0 |  | Total | 349 | 100.0 |
|  |  |  |  |  |  |  |
| *Position* | | |  | *Firm Established* |  |  |
| Senior Management | 81 | 23.2 |  | 1–5 years | 5 | 1.4 |
| Middle Management | 268 | 76.8 |  | 6–10 years | 46 | 13.2 |
| Total | 349 | 100.0 |  | 11–15 years | 176 | 50.5 |
|  |  |  |  | 16–20 years | 88 | 25.2 |
| *Firm Size* |  |  |  | More than 20 years | 34 | 9.7 |
| Medium | 227 | 65.0 |  | Total | 349 | 100.0 |
| Large | 122 | 35.0 |  |  |  |  |
| Total | 349 | 100.0 |  |  |  |  |

**Source:** Author's data analysis

**Table S3.** Full Collinearity Test

| **Variables** | **VIF** |
| --- | --- |
| CSR towards Employees | 3.081 |
| CSR towards Community | 2.703 |
| CSR towards Environment | 2.508 |
| CSR towards Customers | 1.351 |
| Green Organizational Climate | 3.220 |
| Green Shared Vision | 1.259 |
| Workplace Pro-environmental Behavior | 2.264 |

**Source:** Author's data analysis

**Table S4.** Fornell-Larcker criterion

|  | CSRE | CSRC | CSRN | CSRU | GOCL | GRSV | WPEB |
| --- | --- | --- | --- | --- | --- | --- | --- |
| CSRE | **0.760** |  |  |  |  |  |  |
| CSRC | 0.742 | **0.783** |  |  |  |  |  |
| CSRN | 0.686 | 0.677 | **0.736** |  |  |  |  |
| CSRU | 0.361 | 0.287 | 0.391 | **0.768** |  |  |  |
| GOCL | 0.669 | 0.653 | 0.694 | 0.477 | **0.806** |  |  |
| GRSV | 0.284 | 0.182 | 0.177 | 0.011 | 0.228 | **0.795** |  |
| WPEB | 0.595 | 0.445 | 0.478 | 0.257 | 0.662 | 0.433 | **0.790** |

**Note:** CSR towards employees (CSRE), CSR towards community (CSRC), CSR towards environment (CSRN), CSR towards customers (CSRU), Green organizational climate (GOCL), Green shared vision (GRSV), Workplace pro-environmental behavior (WPEB)

**Source:** Author's data analysis

**Table S5.** Heterotrait-monotrait ratio (HTMT) - Matrix

|  | CSRE | CSRC | CSRN | CSRU | GOCL | GRSV | WPEB |
| --- | --- | --- | --- | --- | --- | --- | --- |
| CSRE |  |  |  |  |  |  |  |
| CSRC | 0.838 |  |  |  |  |  |  |
| CSRN | 0.780 | 0.763 |  |  |  |  |  |
| CSRU | 0.436 | 0.345 | 0.476 |  |  |  |  |
| GOCL | 0.736 | 0.710 | 0.765 | 0.561 |  |  |  |
| GRSV | 0.336 | 0.215 | 0.208 | 0.083 | 0.263 |  |  |
| WPEB | 0.677 | 0.501 | 0.542 | 0.310 | 0.730 | 0.512 | - |

**Note:** CSR towards employees (CSRE), CSR towards community (CSRC), CSR towards environment (CSRN), CSR towards customers (CSRU), Green organizational climate (GOCL), Green shared vision (GRSV), Workplace pro-environmental behavior (WPEB)

**Source:** Author's data analysis

**Table S6.** Loading and Cross loadings

|  | CSRE | CSRC | CSRN | CSRU | GOCL | GRSV | WPEB |
| --- | --- | --- | --- | --- | --- | --- | --- |
| CSRE1 | ***0.771*** | 0.561 | 0.524 | 0.215 | 0.472 | 0.247 | 0.449 |
| CSRE2 | ***0.739*** | 0.617 | 0.528 | 0.262 | 0.527 | 0.220 | 0.436 |
| CSRE3 | ***0.752*** | 0.503 | 0.525 | 0.346 | 0.531 | 0.253 | 0.510 |
| CSRE4 | ***0.715*** | 0.566 | 0.495 | 0.232 | 0.460 | 0.226 | 0.449 |
| CSRE5 | ***0.793*** | 0.578 | 0.503 | 0.256 | 0.558 | 0.203 | 0.455 |
| CSRE6 | ***0.782*** | 0.564 | 0.566 | 0.304 | 0.508 | 0.245 | 0.461 |
| CSRE7 | ***0.767*** | 0.562 | 0.510 | 0.298 | 0.491 | 0.117 | 0.406 |
| CSRC1 | 0.600 | ***0.824*** | 0.527 | 0.224 | 0.484 | 0.186 | 0.348 |
| CSRC2 | 0.589 | ***0.765*** | 0.517 | 0.296 | 0.464 | 0.189 | 0.356 |
| CSRC3 | 0.565 | ***0.756*** | 0.488 | 0.197 | 0.529 | 0.131 | 0.352 |
| CSRC4 | 0.587 | ***0.814*** | 0.533 | 0.269 | 0.571 | 0.129 | 0.375 |
| CSRC5 | 0.583 | ***0.759*** | 0.558 | 0.223 | 0.461 | 0.137 | 0.331 |
| CSRC6 | 0.529 | ***0.776*** | 0.554 | 0.196 | 0.509 | 0.136 | 0.328 |
| CSRC7 | 0.618 | ***0.785*** | 0.537 | 0.173 | 0.543 | 0.099 | 0.344 |
| CSRN1 | 0.524 | 0.544 | ***0.762*** | 0.273 | 0.498 | 0.125 | 0.385 |
| CSRN2 | 0.508 | 0.469 | ***0.744*** | 0.290 | 0.478 | 0.137 | 0.336 |
| CSRN3 | 0.547 | 0.443 | ***0.708*** | 0.274 | 0.536 | 0.144 | 0.384 |
| CSRN4 | 0.519 | 0.511 | ***0.740*** | 0.275 | 0.547 | 0.154 | 0.363 |
| CSRN5 | 0.431 | 0.507 | ***0.736*** | 0.311 | 0.516 | 0.086 | 0.333 |
| CSRN6 | 0.443 | 0.449 | ***0.696*** | 0.294 | 0.481 | 0.085 | 0.288 |
| CSRN7 | 0.562 | 0.557 | ***0.759*** | 0.278 | 0.518 | 0.154 | 0.364 |
| CSRN8 | 0.501 | 0.505 | ***0.743*** | 0.310 | 0.507 | 0.151 | 0.354 |
| CSRU1 | 0.323 | 0.244 | 0.290 | ***0.765*** | 0.367 | 0.028 | 0.233 |
| CSRU2 | 0.262 | 0.167 | 0.272 | ***0.758*** | 0.361 | 0.024 | 0.225 |
| CSRU3 | 0.237 | 0.212 | 0.319 | ***0.760*** | 0.334 | -0.043 | 0.126 |
| CSRU4 | 0.283 | 0.254 | 0.320 | ***0.789*** | 0.398 | 0.020 | 0.201 |
| GOCL1 | 0.556 | 0.584 | 0.591 | 0.369 | ***0.832*** | 0.143 | 0.507 |
| GOCL2 | 0.564 | 0.527 | 0.606 | 0.394 | ***0.795*** | 0.159 | 0.520 |
| GOCL3 | 0.531 | 0.529 | 0.546 | 0.393 | ***0.803*** | 0.220 | 0.572 |
| GOCL4 | 0.515 | 0.473 | 0.560 | 0.395 | ***0.812*** | 0.133 | 0.540 |
| GOCL5 | 0.522 | 0.504 | 0.523 | 0.427 | ***0.817*** | 0.225 | 0.557 |
| GOCL6 | 0.583 | 0.569 | 0.574 | 0.387 | ***0.797*** | 0.178 | 0.545 |
| GOCL7 | 0.518 | 0.475 | 0.542 | 0.396 | ***0.794*** | 0.168 | 0.508 |
| GOCL8 | 0.551 | 0.570 | 0.549 | 0.348 | ***0.830*** | 0.132 | 0.539 |
| GOCL9 | 0.509 | 0.501 | 0.544 | 0.351 | ***0.775*** | 0.297 | 0.509 |
| GRSV1 | 0.190 | 0.069 | 0.116 | -0.074 | 0.152 | ***0.824*** | 0.337 |
| GRSV2 | 0.234 | 0.177 | 0.154 | 0.025 | 0.181 | ***0.725*** | 0.321 |
| GRSV3 | 0.202 | 0.140 | 0.121 | 0.049 | 0.197 | ***0.794*** | 0.324 |
| GRSV4 | 0.271 | 0.187 | 0.167 | 0.033 | 0.194 | ***0.834*** | 0.390 |
| WPEB1 | 0.522 | 0.355 | 0.448 | 0.229 | 0.561 | 0.362 | ***0.851*** |
| WPEB2 | 0.431 | 0.323 | 0.281 | 0.135 | 0.481 | 0.380 | ***0.764*** |
| WPEB3 | 0.499 | 0.404 | 0.412 | 0.124 | 0.532 | 0.343 | ***0.799*** |
| WPEB4 | 0.499 | 0.369 | 0.375 | 0.215 | 0.508 | 0.307 | ***0.746*** |
| WPEB5 | 0.433 | 0.333 | 0.412 | 0.241 | 0.538 | 0.269 | ***0.772*** |
| WPEB6 | 0.437 | 0.324 | 0.333 | 0.274 | 0.516 | 0.390 | ***0.804*** |

**Note:** CSR towards employees (CSRE), CSR towards community (CSRC), CSR towards environment (CSRN), CSR towards customers (CSRU), Green organizational climate (GOCL), Green shared vision (GRSV), Workplace pro-environmental behavior (WPEB)

**Source:** Author's data analysis

**Table S7.** PLS Predict

|  | Q²predict | PLS-SEM_RMSE | LM_RMSE | RMSE (Difference) | PLS-SEM_MAE | LM_MAE | MAE (Difference) |
| --- | --- | --- | --- | --- | --- | --- | --- |
| GOCL1 | 0.419 | 0.921 | 0.938 | -0.016 | 0.740 | 0.760 | -0.019 |
| GOCL2 | 0.418 | 0.944 | 0.983 | -0.039 | 0.781 | 0.794 | -0.012 |
| GOCL3 | 0.375 | 0.926 | 0.960 | -0.035 | 0.742 | 0.755 | -0.013 |
| GOCL4 | 0.359 | 0.932 | 0.969 | -0.038 | 0.768 | 0.795 | -0.028 |
| GOCL5 | 0.362 | 0.960 | 0.975 | -0.015 | 0.768 | 0.756 | 0.012 |
| GOCL6 | 0.421 | 0.879 | 0.909 | -0.031 | 0.704 | 0.719 | -0.015 |
| GOCL7 | 0.349 | 0.917 | 0.934 | -0.017 | 0.754 | 0.761 | -0.007 |
| GOCL8 | 0.381 | 0.922 | 0.936 | -0.014 | 0.734 | 0.740 | -0.007 |
| GOCL9 | 0.342 | 0.904 | 0.940 | -0.036 | 0.722 | 0.750 | -0.027 |
| WPEB1 | 0.286 | 1.043 | 1.063 | -0.020 | 0.848 | 0.848 | 0.000 |
| WPEB2 | 0.203 | 1.083 | 1.123 | -0.040 | 0.870 | 0.925 | -0.056 |
| WPEB3 | 0.242 | 1.017 | 1.037 | -0.019 | 0.814 | 0.831 | -0.018 |
| WPEB4 | 0.232 | 1.034 | 1.037 | -0.002 | 0.843 | 0.844 | -0.001 |
| WPEB5 | 0.208 | 1.003 | 1.039 | -0.036 | 0.806 | 0.842 | -0.036 |
| WPEB6 | 0.264 | 1.013 | 1.039 | -0.026 | 0.822 | 0.842 | -0.021 |

**Note:** CSR for Employees (CSRE), CSR for Community (CSRC), CSR for Environment (CSRN), CSR for Customers (CSRU), Green Organizational Climate (GOCL), Green Shared Vision (GRSV), Workplace Pro-environmental Behaviour (WPEB)

**Source:** Author's data analysis

**Table S8.** MICOM Analysis

|  | Original  correlation | Correlation permutation mean | 5.0% | Permutation  p value |
| --- | --- | --- | --- | --- |
| CSRE | 0.998 | 0.999 | 0.998 | 0.144 |
| CSRC | 1.000 | 0.999 | 0.998 | 0.748 |
| CSRN | 0.999 | 0.999 | 0.998 | 0.380 |
| CSRU | 0.994 | 0.996 | 0.989 | 0.177 |
| GOCL | 1.000 | 1.000 | 1.000 | 0.704 |
| GRSV | 0.999 | 0.997 | 0.992 | 0.890 |
| WPEB | 0.999 | 0.999 | 0.999 | 0.285 |

**Note:** CSR towards employees (CSRE), CSR towards community (CSRC), CSR towards environment (CSRN), CSR towards customers (CSRU), Green organizational climate (GOCL), Green shared vision (GRSV), Workplace pro-environmental behavior (WPEB)

**Source:** Author's data analysis
